# Recycling of Carbon Fiber Reinforced Plastic-Containing Waste and Iron Oxide-Containing Dusts as Aggregates in Metallurgical Processes

**DOI:** 10.3390/ma18081838

**Published:** 2025-04-17

**Authors:** Thomas Krampitz, Jan Lampke, Dirk Dietz, Holger Lieberwirth

**Affiliations:** 1 Institut für Aufbereitungsmaschinen und Recyclingsystemtechnik, TU Bergakademie Freiberg, Lampadiusstraße 4, 09599 Freiberg, Germany; holger.lieberwirth@iart.tu-freiberg.de; 2 Haver Engineering GmbH, Halsbrücker Straße 34, 09599 Freiberg, Germany; j.lampke@haverengineering.de; 3 SID Deutschland GmbH, Teinacher Straße 66, 71634 Ludwigsburg, Germany

**Keywords:** CFRP, carbon-fiber for raw material utilization, recycling, pelletizing, agglomeration, metallurgical processes

## Abstract

The recycling of waste materials that are usually expensive to dispose of, such as carbon fiber reinforced plastic (CFRP) dust and ferrous dust or sludge, can open up interesting economic prospects and free up landfill space. The agglomeration process is used to combine these two types of waste and produce an aggregate that can be used in shaft furnaces. The carbon contained in the CFRP dust serves as a potential reducing agent in metallurgical processes. The report shows the technical parameters of the wet agglomeration with subsequent sintering for the production of the pellets and provides evidence of the material recycling of the carbon fiber waste. A comparison with primary pellets shows the suitability.

## 1. Introduction

Carbon fiber reinforced plastics (CFRPs) are used in various lightweight construction applications to reduce moving masses. They are used in the energy sector (wind turbine blades), construction (carbon concrete) and mobility (vehicle, railcar, aircraft construction) [1,2]. The global use of composite materials made of carbon fibers (CFs) has been increasing continuously for years. From 2010 to 2016, the demand for CF almost doubled (2010: 33 thousand tons; 2016: 63.5 thousand tons) [1]. Forecasts predict a continuous increase in demand for CF over the coming years, with annual growth rates of 10–13% [1].

During production and at the end of the product life cycle, waste is generated for recycling. Various recycling processes for CFRP structures are known from the literature, depending on the condition of the waste, whereby a distinction is made between mechanical, chemical and thermal processes. As a rule, the focus is on recovering the fibers [3,4,5,6].

Some CRFP-containing waste is not suitable for fiber recovery. In addition, recycling it again generates waste. In some cases, there are even CFRP-GFRP (glass-fiber reinforced plastics) composites for which no established recycling process yet exists [7]. CFRP-containing dusts are also generated as production waste during the machining of CFRP components in milling or cutting production processes. Some of these are also classified as hazardous. For CFRP-containing waste and dusts without a high probability of carbon fiber recovery, most of the recycling routes proposed in the literature are not applicable.

Due to the poor burnout of CFRP structures and the resulting displacement of the partly unburned CFRPs or CFs into the slag, ash or flue gases, co-incineration in waste incineration plants is not recommended [8,9,10]. CFRP-containing materials must therefore also not be included in mixed municipal waste or incorporated into other material flows [11,12]. The landfilling of untreated waste is prohibited in the EU, with regional exceptions, and strict regulations apply in Germany in particular. Separate collection and separate recycling should be aimed for [13]. With temperatures of >1300 °C and a long residence time, cement production has suitable process conditions for co-incineration of CFRP/CF in principle. However, both the handling of the material flows with the potential release of CFs and the possible discharge of unburned CFs from the combustion chamber into the flue gas treatment prevent their use [14,15]. Alternative disposal routes for thermal utilization are therefore required.

One such alternative utilization is the raw material use of CFRP waste as a reducing agent in metallurgical routes. The carbon in the carbon fiber reduces the iron oxide in iron production and serves as a substitute for primary carbon from coke or similar. The method of converting primary ore, oxidic dusts or sludges back into pig iron in combination with carbon carriers is already known [16]. In the method presented in the report, however, primary raw materials such as coke are replaced by carbonaceous waste such as CFRPs. A safe disposal route is thus presented for CFRPs [17]. However, the influence of the CFRP materials on the properties and behavior of the pellets is to be investigated first. In the presented investigations, the pellets produced are compared with conventional pellets. In this report, primary iron ore fines are used as reference iron oxides.

For the metallurgical processes, process-specific additives must be produced whose granulometric properties, material composition and strength must be adjusted. The CFRP structures must first be milled to finely dispersed powder for pelletizing [18,19]. In contrast, CFRP-containing dusts, with their small particle size, have the advantage that only a reduced effort is required for comminution and processing. The proposed process route would therefore be particularly suitable for CFRP-containing dusts.

## 2. Requirements for CFRPs for Utilization in Metallurgical Processes

### 2.1. Material Requirements

The material properties of the CFRP materials must match the requirements of the metallurgical routes. This includes a sufficient calorific value, maximum carbon content and a minimum content of chlorine-containing chemicals.

As a reference, commercial coke typically has a calorific value of between 28 and 30 MJ kg^−1^, depending on the quality, with a carbon content of over 92%. These values apply to dry material without significant moisture. The chlorine content is generally very low, typically less than 0.1%. Coal usually contains little chlorine. For this reason, the chlorine values in coke are usually not specified [20].

For comparison, Table 1 shows the results of a combustion analysis of various CFRP-containing samples from selected waste sources with different material compositions and CFRP contents. The CFRP-O sample serves as a reference material and is produced with a defined material composition and specific structural design for the tests. The samples CFRP-A and CFRP-B originate from vehicle construction and were cut out of load-bearing structural parts. The samples CF-T and CF-K are different reinforcement bars from the production of carbon concrete. The last two samples come from a wind turbine blade built in 2019: one sample consists of laminate with alternating layers of CFRP and GFRP from the tip of the wind turbine, the other from the solid GFRP structure from the base of the wind turbine. The measured values were determined using standardized analysis methods and can be found in the DIN standards listed. All CFRP materials examined have a sufficiently high calorific value and high carbon content compared to coke. CFRP materials with an epoxy resin matrix exhibit minor fluctuations. CFRPs with a thermoplastic matrix have slightly lower carbon contents. Combination textiles made from a fiber mixture of CFRPs and glass fiber reinforced plastics (GFRPs) are significantly lower in carbon content and calorific value compared with pure CFRPs. Mixtures of carbon and glass fibers therefore have significantly lower values. GFRPs with an epoxy resin matrix have a very low carbon fiber content and calorific value, resulting in a clear difference to CFRPs. The use of GFRPs must therefore be distinguished from CFRPs. The lower the CFRP content, the lower the calorific value and carbon content, thus limiting its utilization in metallurgy. With the exception of the CFRP-B sample, the chlorine content was unremarkable. The investigation showed that chlorine chemicals can be present in CFRPs depending on the type of epoxy resin or the use of flame retardants.

Waste containing CFRPs should first undergo treatment to separate materials that are harmful to the process, such as metals or plastics with halogens. However, certain impurities cannot be completely eliminated. New developments that improve the functional advantages of lightweight structures can also have harmful effects on recycling. For example, phosphorus-containing flame retardants are introduced into CFRPs to minimize the release of CFs in the event of a fire [21]. In this respect, the waste streams must be subjected to permanent monitoring with regular analysis of the material composition in order to limit the accumulation of process-related pollutants in the recycling route.

### 2.2. Process Requirements for the Thermal Conversion of CFRP

Process requirements and respective solutions are presented in Table 2.

Table 3 shows a selection of potentially suitable and relatively robust metallurgical processes for CFRP-containing waste. Advantages and disadvantages can be found in [14,22,23]. Therefore, at least in Europe, for cost reasons, only the electric arc furnace route for the production of steel and the shaft furnace route for the production of pig iron [15,24] remain for CFRP waste utilization in combination with ferrous raw materials. The utilization of CFRP materials should be regarded as recycling, with the carbon serving as a reducing agent.

Electric arc furnace for steel: At temperatures of over 1600 °C and for a residence time of approx. 40 min, the CFRP materials are placed in the furnace and brought into contact with the liquid metal phase during the melting of the metals. The CFRP structures react on the surface and decompose layer by layer from the outside inwards. The CFRP material acts as a reducing agent for iron oxides and replaces coke in the process [15,25]. In order to achieve the greatest possible effect, CFRP pieces are introduced into the stationary liquid slag phase [26]. The use of CFRP materials as reinforcing material in briquettes has proven its worth for improved feeding. The advantage of this application is that the mechanical and thermal stability of the briquettes increases, and the additives can be dosed more precisely in the furnace. The application of recipes for the briquettes also allows the targeted production of additives [27,28]. The CFRP is crushed to <5 mm, or dusts are used for this purpose.

Shaft furnace for pig iron: The shaft furnace is an important component in the production of pig iron. In this furnace, typically, coke and iron are reacted at high temperatures of over 1400 °C and a residence time of around 12 h in a reducing atmosphere. However, CO_2_ is generated. In the new approach, CFRP is used as a reducing agent to replace the coke. The CFRP-containing residues are either shredded to <1 mm or CFRP dust is used and then pelletized together with iron ore or iron oxide dust [29]. The process is suitable for recycling two groups of waste that are difficult to dispose of: iron-containing grates, dusts or scale on the one hand and CFRP-containing residues and dusts on the other. The advantage lies in the fact that the reaction partners with a large specific surface area are brought together directly in a pellet [18]. This approach is to be pursued further in the report.

## 3. Requirements for CFRP Iron Pellets for Use in Shaft Furnaces

In order to avoid the release and thus the discharge of free carbon fibers, the CFRP cannot be used as loose fill. Suitable aggregates or pellets with defined properties must be produced in which the CFRP is bound and whose properties correspond to those of iron ore pellets. Table 4 lists the most important requirements for sintered pellets that allow economical use in shaft furnaces [30,31].

Particle size: Although pellets with a smaller pellet diameter have a larger surface-to-volume ratio, the flow and reaction cannot be controlled in the shaft furnace if the diameter is too small [31,32]. Papcek defines the minimum diameter as d_min_ > 8.65 mm. The upper limit of the pellet size is determined by the decreasing reducibility at around 16 mm. Larger pellets have worse gas penetration and a higher sinking speed in the burden column. The requirement of a uniformly reduced state at the beginning of the melting zone justifies a largely monodispersity of the pellets [31]. In practice, a particle size distribution of between 10.4 and 12.5 mm is aimed for [33,34], as specified in ISO 4700 [35].

Strength parameters: For the standardized compressive strength (ISO 4700 [35]) of a single, sintered pellet, guide values for σ_Ed,B_ of between 1500 and 2000 N are given [36,37,38]. The tumble index (TI) and the abrasion index (AI), according to ISO 3271 [39], are determined by stressing the pellets in a rotating drum. The sieve residue at 6.3 mm defines the tumble index and the sieve passage at 0.5 mm defines the abrasion index. Values greater than 95% are preferred for TI (>6.3 mm) and values less than 5% for AI (<0.5 mm) [36,37].

Pellets with a carbon carrier such as CFRP-GFRP mixtures as an integrated reducing agent must be produced accordingly for the shaft furnace.

## 4. Experimental Materials

### 4.1. CFRP

The tests were carried out with two different epoxy resin-based CFRP laminates. The reference material (designation: “CFRP-O”) was a laminate from the manufacturer Carbon-Werke Weißgerber, which was available as sheet material and contained three layers of the UD prepreg PREDO PR-DU 600/1250 FT109 33 in 0°/90°/0° orientation. The prepreg consists of the roving type SIGRAFIL CT50-4.0/240-E100 and the resin system FT109, with a glass transition temperature of approx 120 °C. A complex-shaped component made from production waste from a vehicle manufacturer (designation: “CFRP-A”) serves as a reference material. The component consisted of a multilayer prepreg fabric with a 0° to 90° orientation, the matrix material epoxy resin with a glass transition temperature of 120 °C and an unknown fiber type. The material data are summarized in Table 5. The density of the composite material is calculated from the combined density of both components. With the values for the C-fibers of 1.8 g cm^−3^ and epoxy resin of 1.2 g cm^−3^, a mixed density of 1.55 g cm^−3^ is derived for the composite material CFRP-O and a value of 1.50 g cm^−3^ for CFRP-A. The mass fractions can be found in Table 1 and Table 5.

To pelletize the CFRP materials, the large-area structures first had to be comminuted to a particle size < 100 µm using a two-stage comminution process. The procedure is described in [18]. Despite sieving with 100 µm aperture, particles with edge lengths of up to 250 µm are contained, which is due to the elongated particle shape of the carbon fibers. Compared to bentonite, CFRP has a similarly high specific surface area. The bulk density of CFRPs is comparatively low. In order to influence the resulting density of the pellets as little as possible to prevent floating, only small mass fractions of CFRPs are permissible for pelletizing. The stoichiometry of carbon to iron oxide is therefore a resulting variable from the requirements of the pellet properties and was not specifically adjusted during the tests in accordance with the chemical requirements.

### 4.2. Magnetite Iron Ore

The iron oxide used for the tests was a primary iron ore from Norway (magnetite ore) with a content of 68.2% Fe, 0.2% CaO, 0.21% MgO and 0.28% Al_2_O_3_. The most important physical parameters of the feedstock are shown in Table 6.

### 4.3. Bentonite

Bentonite from Greece with a content of approx. 61% SiO_2_, 20% Al_2_O_3_, 3% Na_2_O, 0.2% CaO and 3% Fe_2_O_3_ was used for the investigations. The application of bentonite in iron ore pelletizing has several positive influences on relevant pellet characteristics due to its hygroscopic properties and swelling capacity [36] (p. 110 ff). Sintered pellets with bentonite have a lower tendency to abrade, and respectively, a lower abrasivity index (AI).

## 5. Stoichiometric Considerations

The overall reaction takes place in several stages, whereby Fe_3_O_4_ is gradually reduced to metallic iron. The simplified reaction equation is Fe_3_O_4_ + 4 C → 3 Fe + 4 CO.

As 4 moles of carbon are stoichiometrically required for the conversion of 1 mole of Fe_3_O_4_, the following approximate calculation results with the corresponding molar mass of M_Fe3O4_ = 231.55 g mol^−1^ and M_C_ = 12.01 g mol^−1^. The magnetite ore contains 90.4% magnetite. This results in the following amount of material n_Fe3O4_ for 100 g magnetite ore:

$$n_{{Fe}_{3}O_{4}}=\frac{90.4\;g}{231.55{gmol}^{-1}}=0.39\;mol$$

The following amount of carbon *n_C_* is obtained for the stoichiometric conversion:

$$n_{C}=4\cdot 0.39\;mol=1.56\;mol$$

This results in the following mass of carbon *m_C_:*

$$m_{C}=1.56\;mol\cdot 12.01\;g{mol}^{-1}=18.75\;g$$

For the complete stoichiometric conversion of 100 g magnetite ore with a content of 90.4 g magnetite to pig iron, approx. 18.7 g carbon would be required. As the maximum content of CFRP is 5% (of which approx. 96% is carbon), this is only sufficient for partial reduction. The reduction of magnetite (Fe_3_O_4_) to hematite (Fe_2_O_3_) is a process in which less carbon is required. Instead, only part of the oxygen is removed from Fe_3_O_4_. The simplified reaction equation is Fe_3_O_4_ + C → 3 Fe_2_O_3_ + CO

The following amount of carbon *n_C_* is obtained for the stoichiometric conversion:

$$n_{C}=n_{{Fe}_{3}O_{4}}=0.39\;mol$$

This gives the following mass of carbon *m_C_*:

$$m_{C}=0.39\;mol\cdot 12.01\;g{mol}^{-1}=4.66\;g$$

**Figure 1:**
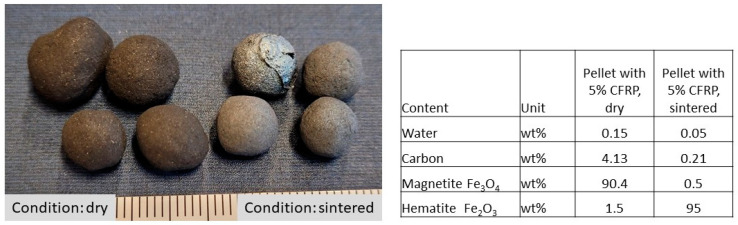
Pellets containing 5% CFRP and their analysis results according to DIN 51733.

For the reduction of 90.4 g of magnetite to hematite, only approx. 4.6 g of carbon is required. According to Figure 1, the pellets containing 5% CFRP with approx. 96% carbon content contain approx. 4.8 g of carbon. This means that the use of CFRP in pellets is largely sufficient, at least for the stage of reduction to hematite.

Analyses of the sample with a CFRP content of 5% in accordance with DIN 51733 [40] confirm the reduction of the carbon content from 4.13 wt.% in the dried state to 0.21 wt.% in the sintered state (Figure 1). The consumption of carbon was thus proven.

The magnetite content decreases from 90.4% to below 0.5%, with a simultaneous increase in hematite from 1.5% to over 95%, which proves the reducing effect of the carbon from the CFRP structures according to the stoichiometric calculation.

## 6. Agglomeration of Iron Ore Pellets by Pelletizing

### 6.1. Agglomeration by Pelletizing

Pellets are produced by increasing the particle size of finely dispersed starting materials by means of agglomeration (pelletizing). Individual particles or smaller agglomerates accumulate by rolling or rolling motion. Primarily capillary binding mechanisms are activated, which are greater than the existing separating forces. This results in the so-called green pellet [33,41,42,43]. When pelletizing iron ore, green pellets are first produced while still moist and are then dried. Drying activates stronger adhesive forces. Finally, the dry pellet is sintered by gradually increasing the temperature with firmly defined holding points up to 1290 °C [36,44]. The basic principles and mode of operation of agglomeration can be found in [33,41,42,43].

### 6.2. Parameters Influencing the Pelletizing Process

The agglomeration takes place on a pelletizing table rotating around an inclined axis [33,43].

Process-related influencing variables: The position of the material and liquid feed on the pelletizing disc has an influence on the size of the pellets [45,46]. The inclination angle of the pelletizing table axis, α, should be approx. 45° and the operating speeds, n_B_, should be between 0.72 and 0.79 n_crit_, respectively [47,48], with n_crit_ being the critical speed at which the material starts to centrifuge on the table. The amount of feed material per time unit depends on the throughput at the table, which increases with increasing table diameter. The dependency is calculated using the pelletizing factor of iron ore with 1.6 t/(m^2^h) multiplied by the diameter of the plate squared [34,42,49].

Design-related influencing variables: The table diameter, D_T_, the table rim height, H_T_, and the angle of inclination, α, influence the mass of material on the table and thus the residence time, t_V_, of the material as well as the contact pressure on the fines [43]. Larger pelletizing table diameters are therefore preferred for increased process stability.

Material-related influencing variables: As the particle size of the feed material decreases, the mass-related specific surface, S_m_ (in cm^2^ g^−1^), increases. According to Meyer, this leads to the progressively increasing individual compressive strength of the pellets [36] (p. 102). Furthermore, moisture has a considerable influence on the pellet size as well as on the porosity and strength of the pellets. A liquid content of 6 to 14 % by weight is specified as optimal for pellet formation [36,50,51,52].

### 6.3. Experimental Design

Conclusion for the experiments: As a result, the feed materials iron ore, bentonite and CFRP should be as finely ground as economically feasible, with a high specific surface area. The moisture is adjusted by adding water. The experiments are carried out in the technical center jointly operated by Haver Engineering GmbH (HEM) and the IART on a PT400 laboratory pelletizing table with table diameter D_T_ = 400 mm, edge height H_T_ = 95 mm, n_crit_ = 0.4, axis inclination angle α = 35°. Before pelletizing, the components are mixed in an intensive mixer. After pelletizing, drying at 105 °C and sintering up to 1290 °C takes place. These experimental parameters remain constant throughout the tests.

Eight different batches, I–VIII, are prepared, with a constant mass of 100 g magnetite ore and 2% water, according to the following:
➢batch Iapprox. 2 wt.% bentonite added,➢batches II–VIapprox. 2 wt.% bentonite and 0.5 to 10 wt.% CFRP-O added,➢batches VII–VIIIapprox. 2 wt.% bentonite and 0.5 to 1 wt.% CFRP-A added.

Table 7 shows the resulting material composition and mass distribution.

## 7. Comparison of the Characteristic Properties of the Sintered Pellets

**Figure 2:**
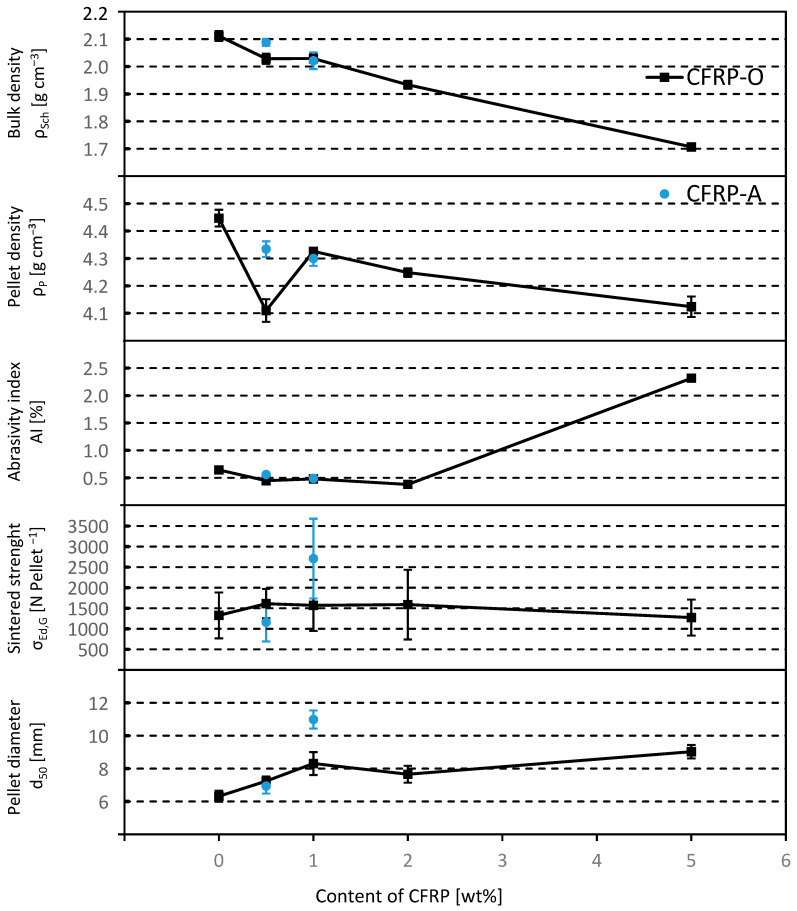
Comparison of the characteristic values of the sintered pellets as a function of the CFRP content (values with standard deviation, according to [53]).

Pellets with a CFRP content larger than 5 wt.% (batch II) exhibits too much melting on the surface, so that no sintered pellets could be produced with the initially specified regime. Only pellets with a CFRP content of up to 5 wt.% were considered for the following conclusions according to Figure 2.

(a) The diameter of the pellets produced reaches between 6 and 11 mm. It tends to increase with increasing CFRP content. Only the mixture with 1 wt.% CFRP-A results in pellets matching the target size range. The other pellets produced in the tests tend to be too small for utilization in the shaft furnace. One reason for this is probably the relatively small laboratory pelletizing table. It can be assumed that larger pellets can also be produced if industrial scale pelletizing tables are used.

(b) The strength [N] remains almost constant across all CFRP contents. A significantly higher value can only be achieved with a setting of 1 wt.% CFRP-A.

(c) The abrasion resistance, AI, remains constant up to a content of approx. 2 wt.% CFRP and then increases to AI > 2% with 5 wt.% CFRP-O content. All values remain in the target range below 5%. The drum strength, TI, is over 99.5% and therefore well above the required value of 95%.

(d) The pellet density decreases with increasing CFRP content. The mixture with 1 wt.% CFRP-O is evaluated as an outlier. This may be due to the increasing porosity that forms as a result of the transformation of the CFRP materials and the newly formed pores and cavities.

(e) As expected, the bulk density also decreases with increasing CFRP content. The reference mixture without CFRP achieves the highest bulk density.

**Figure 3:**
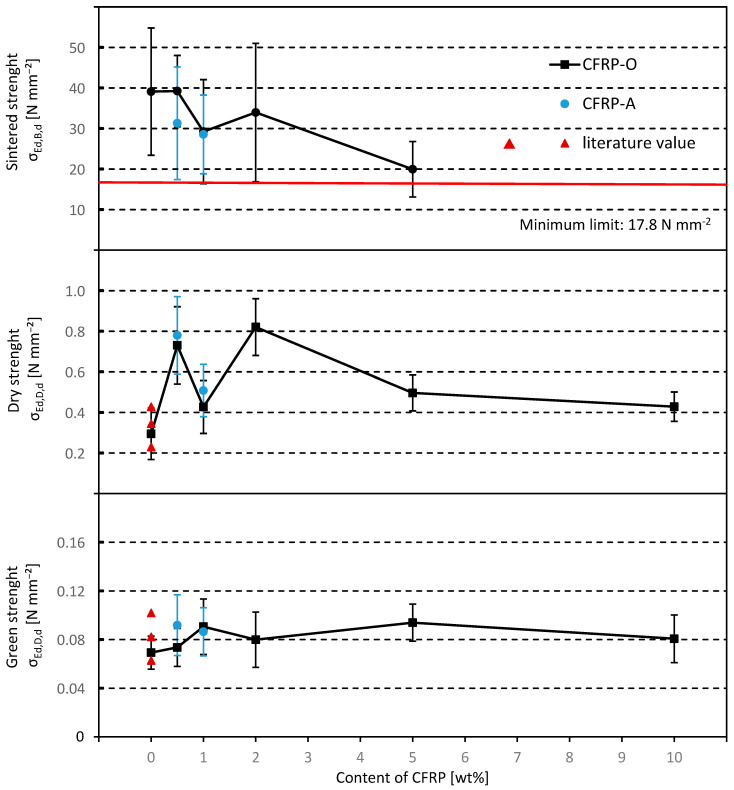
Comparison of the diameter-related strengths as a function of CFRP content, literature source [36] and process condition of the pellets (according to [53]).

In order to better illustrate the influence of the CFRP, the characteristic values were normalized as follows. As a guide value for the sintered pellets, the minimum strength of 2000 N per pellet to be achieved is related to the circular cross section resulting from the average pellet target diameter in the range of 11.4 to 12.5 mm (d_50_ = 11.95 mm), according to ISO 4700 [35]. This results in a minimum value of 17.8 N mm^−2^, which is entered graphically in the diagram. The cross-section related strengths, σ_Ed,X,d_, in [N mm^−2^] are shown in Figure 3.

The addition of CFRPs increases the green strength of the pellets slightly and the dry strength significantly. The addition of CFRPs therefore has clear advantages for handling the pellets. The strength of the sintered pellets, on the other hand, decreases. This can be explained by the consumption of carbon during the chemical reaction, whereby cavities in the form of pores are created in the pellet and thus lead to weak points in the pellet. However, porous structures can in turn have a positive effect on the process control in the shaft furnace, which was not investigated here. The green and dry strengths of the pure iron ore pellets in the presented tests are consistent with the values of Meyer [36] and confirm the results.

## 8. Structure of the Pellets

**Figure 4:**
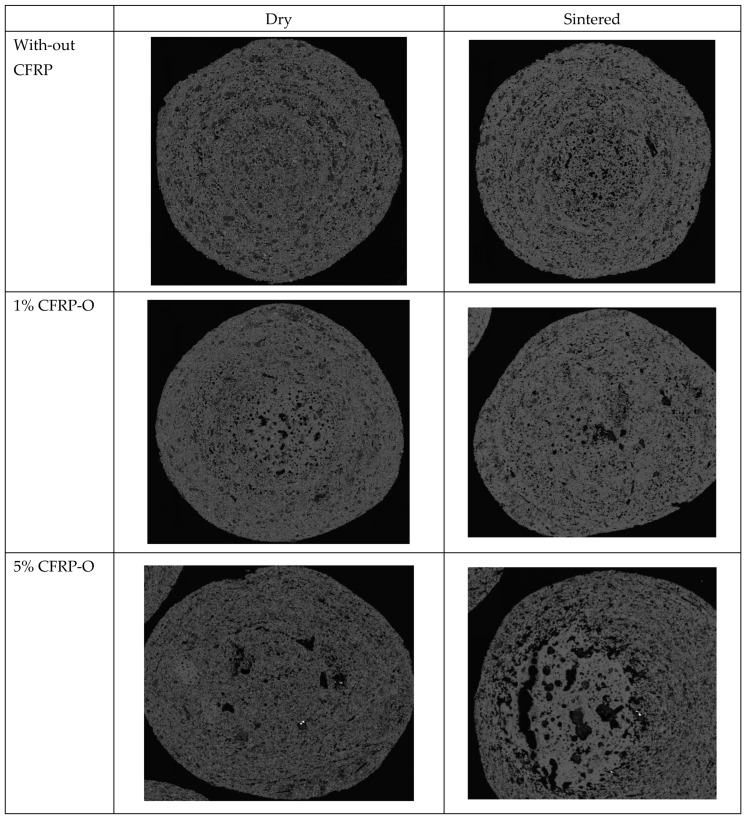

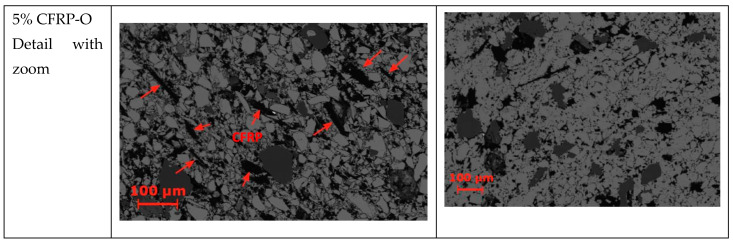
Thin section of pellets with different CFRP contents and conditions.

For the microstructural analysis of the pellets, thin sections were prepared and examined using a scanning electron microscope (SEM). Figure 4 shows pellets with various carbon fiber contents in the dry and sintered state. The basic mechanism of pellet formation by means of build-up agglomeration is clearly shown by the visible snowball structure. A germination zone can be seen in the middle of the pellets, which grows outwards with increasing residence time on the pelletizing plate due to layer-by-layer deposition. The light gray grains indicate magnetite, the dark gray grains indicate bentonite. With increasing CFRP, the pore size and pore permeability increase. This explains the reduction in strength and density.

For the microstructure analysis of the pellets, thin sections were prepared and examined using a scanning electron microscope (SEM). Figure 4 shows pellets with different carbon fiber contents in the dry and sintered states. The basic mechanism of pellet formation by means of build-up agglomeration is clearly shown by the visible snowball structure. A germination zone can be seen in the middle of the pellets, which grows outwards with increasing dwell time on the pelletizing plate due to layer-by-layer deposition. The light gray grains indicate magnetite, the dark gray grains indicate bentonite. With increasing CFRP, the pore size and pore permeability increase, which explains the reduction in strength and density.

## 9. Summary

The tests proved that iron ore pellets with a content of approx. 2 wt.% bentonite and up to 10 wt.% carbon-fiber reinforced polymer (CFRP) can be produced.

The investigation showed the effectiveness with two different CFRP materials. The potential usability of CFRPs was thus verified. Under the selected experimental conditions, an increase in the CFRP content resulted in a tendency for the average pellet diameter to increase. The increased addition of CFRPs to the iron oxide at more than 5 wt.% led to a reduction in the melting temperature and to melting of the pellet surface. At the required sintering temperature of 1290 °C, the melting was too strong and the pellets caked together and were no longer separated. The amount of CFRPs added should therefore be limited to less than 5 wt.%.

The process parameters selected for the laboratory pelletizing table did not meet the requirements for the average pellet diameter in full. The best results were achieved with a CFRP content of 0.5–1 wt.%. However, it should also be possible to produce larger pellets by using a pelletizing table with a larger diameter.

The practical assessment of the melting and reduction behavior of the sintered pellets under real conditions in the shaft furnace, in particular with regard to temperature and pressure, is still pending. It can be assumed that only the use of low CFRP contents (0.1–0.5 wt.%) will be suitable in the shaft kiln process, as hardly any changes to the operating mode, such as the charging method and the complete melting process, are permitted during operation of the shaft kiln.

The required green and dry pellet strengths were achieved. However, the content of 5% by weight of CFRPs should not be exceeded here either due to the increase in pore formation and thus the reduction in strength. When dried, the CFRP materials act like a fiber reinforcement and lead to a higher pellet strength. This results in significant advantages for handling the pellets during processing, as there is less abrasion or breakage. The process costs drop as a result. In the sintered state, the addition of CFRP materials does not result in any significant advantages or disadvantages in terms of strength. Energy-related advantages or disadvantages resulting from the addition of CFRPs must be examined.

In conclusion, the addition of CFRPs in the shaft furnace process represents an option for recycling CFRP-containing waste that also does not involve the exposure of fibers.

Around 23.7 million tons of pig iron were produced in Germany in 2022, with the majority of this quantity being produced from primary pig iron using the classic shaft furnace process [54]. With an approximate ratio of 1.5 tons of iron ore to 1 ton of pig iron, around 175,000 t a^−1^ of CFRPs could theoretically be safely recycled in Germany if 0.5 wt.% of CFRPs were added to the pig iron.

Since the processing of the ore into iron ore pellets usually takes place close to the primary raw material deposit and not in Germany, the transportation of CFRP waste over long distances can be ruled out for cost reasons alone, and the application of the proposed mixture for pellets from primary ores is rather unlikely. However, there are large quantities of iron oxides in Germany or Europe as a waste product of iron processing, the chemical industry or steel processing. The processing of iron-containing oxides in combination with carbon fiber-containing dusts opens up very interesting possibilities for the recycling of iron waste with simultaneous utilization of CFRP waste.

The article refers to laboratory tests and experiments. For a detailed evaluation of the behavior of the pellets under real operating conditions in the shaft furnace or other metallurgical processes, further tests should be carried out under realistic conditions. It is also necessary to determine how the addition of CFRPs affects the long-term stability of the pellets and the overall operating parameters.

The requirements for the pellets as feed material for the shaft furnace are known and described in the literature. It is assumed that if the strength values are complied with, then the pellets can also be used. The proof of the sufficient strength of the pellets in connection with the utilization of the carbon fibers in the process refute the greatest concerns for large-scale implementation and support further research.

## Figures and Tables

**Table 1:** Analysis results of certain CFRP-containing materials.

Type			CFRP-O	CF-O	CFRP-A	CFRP-B	CF-T	CF-K	CFRP/GFRP	GFRP
Description			0/90/0 prepreg		Vehicle manufacturer A	Vehicle manufacturer B		Combinated textile	Combinated prepreg	Wind turbine blade
Resin type			Epoxy	-	Epoxy	Epoxy	Thermo-plast PA6	Epoxy	Epoxy	Epoxy
Parameters	Unit	Method	Value	Value	Value	Value	Value	Value	Value	Value
Carbon	wt.-%	DIN 51732	86.6	94.8	87.0	91.7	73.5	68.8	64.5	29.7
Calorific Value Hu	kJ kg^−1^	DIN 51900	30,600	31,900	31,400	31,300	29,300	24,500	25,900	11,600
Chlorine	wt.-%	DIN 51727	0.18	0.01	0.04	0.47	0.01	0.02	0.03	0.15

**Table 2:** Requirements for CFRP utilization.

*Requirements*	*Process Management*
Complete combustion	Longer dwell time and higher ambient temperatures (>800 °C) compared to conventional waste incineration plants
Reducing atmosphere	High carbon content and calorific value for the chemical reduction system
Minimal exposure of fibers in the process	Largely closed process chamber with the greatest possible avoidance of fiber discharge into the exhaust air Favoring the use of liquid phases at high temperatures and high residence time
Tolerance to material mixtures	CFRP-containing waste is mostly combined with other materials or contains residual adhesions; therefore, a high tolerance to GFRP, other fiber reinforcements and low quantities of metallic materials or halogens is required

**Table 3:** Potential metallurgical routes for CFRP utilization.

	Electric Arc Furnace			Shaft Furnace
Product	Steel	Calcium carbide	Silicon carbide	Pig iron
Temperature	Ca. 1600 °C	>2000 °C	>2000 °C	Ca. 1500 °C
Residence time	Ca. 40 min	hours	no indication	>12 h

**Table 4:** Requirements for sintered iron ore pellets and advantages in the shaft furnace.

Requirement	Advantage
Narrow particle size distribution with high permeability High pressure and abrasion resistance at high temperature High reducibility (high carbon content) Low swelling capacity or reduction softening	No segregation in the furnace Good gas flow through the pellets Increase in indirect reduction

**Table 5:** Characteristics of the CFRP feed material.

CFRP-O		CFRP-A
Laminat PREDO PR-DU 600/1250 FT1091 33	Roving SIGRAVIL C T50-4.0/240 E100	
Mass of resin [%] 33	Fiber sizing Epoxy	Mass of resin [%] ca. 39.3 +/− 1.1 ^(a)^
0°—Tensil strenght [MPa] 1900	Tensile strength [MPa] 4000	
0°—Elongation-modulus [GPa] 130	Elongation modulus [GPa] 240	
0°—Elongation at break [%] 1.2	Filament diameter [µm] 7	
Interlaminate shear strength [MPa] 64	Amount of filament in Roving 50,000	
Wall thickness [mm] 1.9		Wall thickness [mm] 1.7

^(a)^ Empirically determined value: muffle furnace Nabertherm L15/13/C450-LT, 30 min residence time at 500 °C.

**Table 6:** Properties of the magnetite ore, bentonite, CFRP-O and CFRP-A.

Property	Unit	Analytical Method	Magnetite Ore	Bentonite	CFRP-O	CFRP-A
Particle size < 56 µm	%	DIN EN ISO 8130-13	88.6	83.1	-	-
Particle size x_50_	µm		23.3	30.6	24	19
Particle size x_90_	µm	DIN EN ISO 8130-13	58.4	64.7	101	87
Fiber length x_90_	µm		-	-	222	254
Moisture content	%	ISO 10251	8.2	7.3	0.4	1.9
Specific surface S_m_ according to Blaine	cm^2^ g^−1^	DIN 66126	2095	8586	6621	6681
Bulk density dry	g cm^−3^	DIN ISO 693	2.26	0.86	0.29	0.24

**Table 7:** Composition of the tested pellets.

Batch	Magnetite Ore	CFRP-O	CFRP-A	Bentonite	Water	Magnetite Ore	Total	CFRP-O	CFRP-A	Bentonite	Water	Total
	g	g	g	g	g	g	%	%	%	%	%	%
I	100	0	-	2	2	104	96.2	0.0	-	1.9	1.9	100
II	100	10	-	2	2	114	87.7	8.8	-	1.8	1.8	100
III	100	1	-	2	2	105	95.2	1.0	-	1.9	1.9	100
IV	100	5	-	2	2	109	91.7	4.6	-	1.8	1.8	100
V	100	2	-	2	2	106	94.3	1.9	-	1.9	1.9	100
VI	100	0.5	-	2	2	104.5	95.7	0.5	-	1.9	1.9	100
VII	100	-	1	2	2	105	95.2	-	1.0	1.9	1.9	100
VIII	100	-	0.5	2	2	104.5	95.7	-	0.5	1.9	1.9	100

## Data Availability

Data is contained within the article.
